# The modified 5-item frailty index as a predictor of perioperative risk in patients undergoing percutaneous nephrolithotomy

**DOI:** 10.1007/s11255-024-04178-3

**Published:** 2024-08-01

**Authors:** Kays Chaker, Yassine Ouanes, Mahdi Marrak, Nader Gharbia, Moez Rahoui, Boutheina Mosbahi, Mokhtar Bibi, Wassim Ben Chedly, Yassine Nouira

**Affiliations:** 1 Urology Department, LA RABTA Hospital, University of Tunis El Manar, Bab Saadoun, 1006 Tunis, Tunisia; 2 Anesthesiology Department, LA RABTA Hospital, University of Tunis El Manar, Tunis, Tunisia

**Keywords:** Percutaneous nephrolithotomy, Stone, Frailty, Complications

## Abstract

**Introduction:**

The modified 5-item frailty index is a relatively new tool to assess the post-operative complication risks. In urology, there is limited literature on the impact of frailty on percutaneous nephrolithotomy (PCNL) outcomes. We aimed to compare the predictive value of the modified 5-item frailty index (mFI-5) to identify high risk patients prior to PCNL.

**Methods:**

A database of patients undergoing PCNL, between 2015 and 2022, was analyzed. Patient frailty was assessed using the mFI-5 index. The mFI-5 index was calculated based on the presence of the five co-morbidities: congestive heart failure within 30 days prior to surgery, diabetes mellitus, chronic obstructive pulmonary disease, partially dependent or totally dependent functional health status at time of surgery, and hypertension requiring medication. Patients were grouped as not frail (mFI-5 = 0), intermediate (mFI-5 = 1), and severely frail (mFI-5 ≥ 2). Primary outcomes were 30-day postoperative complications. Secondary outcomes were hospitalization: total hospital length of stay, reoperation, and unplanned readmission.

**Results:**

From a total of 320 PCNL patients included for analysis, 54.06% (*n* = 173) were not frail, 17.81% (*n* = 57) were intermediate, and 28.12% (*n* = 90) were severely frail. Frail patients were likely to be older (*p* = 0.002) and have a higher American Society of Anesthesiologists score (*p* = 0.001), chronic kidney disease (*p* < 0.001). Patients of intermediate or severe frailty were more likely to exhibit postoperative sepsis (*p* = 0.042), significant blood loss (*p* = 0.036) and require intensive care units admissions (*p* = 0.0015). Frail patients had a longer hospital length of stay (*p* < 0.001) and tended to require reoperation (*p* = 0.001), and unplanned readmission (*p* = 0.02).

**Conclusion:**

Frailty assessment appears useful in stratifying those at risk of extended hospitalization, septic and hemorrhagic complications, readmission, or reoperation after PCNL. Preoperative assessment of frailty phenotype may give insight into treatment decisions and assist surgeons in counselling patients on expected course and hospital stay following PCNL.

## Introduction

In 2017, the modified frailty index-5 (mFI-5) was proposed by identifying the five most consistently reported variables from the mFI-11 [1]. The mFI-5 is a simple scoring system. Despite its recent introduction, it is renowned for being easily usable in daily clinical practice. The mFI-5 used to assess frailty and biological age, has proven to be a reliable predictor of complications and mortality in various surgical specialties [2]. In urology, there is limited literature on the impact of frailty on percutaneous nephrolithotomy (PCNL) outcomes. The PCNL has always been described as a minimally invasive technique, due to the fact that, unlike open surgery, it limits the operative trauma [3]. However, experience has shown that it is not devoid of complications, which can be both frequent and serious, both in terms of functionality and vital aspects. We aimed to compare the predictive value of the mFI-5 to identify high risk patients prior to PCNL.

## Patients and methods

A database of patients undergoing PCNL, between 2015 and 2021, was analyzed clinical and intra-operative data including, demographics, relevant labs, number of punctures, stone location, and stone characteristics were analyzed. Patient frailty was assessed using the mFI-5 index, which is assessed between 0 and 5 for the cumulative presence of the five components. The mFI-5 index was calculated based on the presence of the five co-morbidities: congestive heart failure within 30 days prior to surgery, insulin-dependent or noninsulin-dependent diabetes mellitus, chronic obstructive pulmonary disease need on regular treatment, partially dependent or totally dependent functional health status at time of surgery, and hypertension requiring medication [4]. The mFI-5 was calculated by assigning 1 point for each comorbidity [4]. Patients were grouped as not frail (mFI-5 = 0), intermediate (mFI-5 = 1), and severely frail (mFI-5 ≥ 2). 30 days post-operative complication and resource utilization (including length of hospital stay, ancillary procedure and secondary readmission) in the management of PCNL were evaluated. Significant blood loss was operationalized by a pre- vs. post-operative hematocrit level decrease greater than 5%. Post PCNL infectious complications were classified into 3 categories: Urinary tract infection, sepsis, and septic shock. Urinary tract infection was defined as the presence of a body temperature ≥ 38.5 °C within 48 h after surgery and the presence of bacteriuria within one week [5]. Sepsis was defined as a qSOFA (rapid sepsis-associated organ failure assessment) score ≥ 2 of the following scoring criteria: respiratory rate ≥ 22/min; altered mental status (Glasgow Coma Score ≤ 13); and systolic blood pressure ≤ 100 mmHg [6, 7]. Septic shock is defined by the combination of: sepsis with hypotension not responding to adequate fluid resuscitation, requirement for vasopressor drugs to maintain a Mean Arterial Pressure (MAP) ≥ 65 mmHg, Lactate levels > 2 mmol/l [8].

### Statistics

An analysis of all these data were carried out using IBM SPSS Statistics 25 software. The results of quantitative variables were presented as mean ± standard deviation (SD) or median and interquartile range (IQR), and those of qualitative variables as number and percentage. For normally distributed variables, the t-test was used to compare two means, and analysis of variance (ANOVA) was used to compare several means. For categorical variables, the chi-square test and Fisher's exact test were used in independent samples. The Spearman coefficient was used to assess the strength and direction of monotonic relationships between variables. A p value of < 0.05 was considered statistically significant.

## Results

We included 320 patients. The mean age of the patients was 49 ± 13 years. The mean stone size was 28.40 ± 10.73 mm. The mean density of the stone was 1011.87 ± 293.61 UH. From these patients, 54.06% (*n* = 173) were not frail, 17.81% (*n* = 57) were intermediate, and 28.12% (*n* = 90) were severely frail. Table 1 lists the clinicopathological characteristics of patients. Frail patients were likely to be older (*p* = 0.002) and have a higher American Society of Anesthesiologists score (*p* = 0.001), chronic kidney disease (*p* < 0.001). Thirty-five patients (11%) were identified with positive preoperative urine cultures. These patients received antibiotic treatment tailored to the results of the antibiogram, with urine sterilization before the operation. All patients received antibiotic prophylaxis 2 h before induction, consisting of 2g of Cefazolin. All procedures were performed in the prone position. The combined approach was not used in any case. Kidney puncture is performed under fluoroscopic guidance. This fragmentation energy uses compressed air in a handle, which is transmitted through a metal probe like a « jackhammer». Preoperative drainage with a double J stent was noted in 136 patients (42.5%). It was indicated in 86 patients (63.2%) for obstructive acute pyelonephritis, in 30 patients (22%) for obstructive calculous anuria, and in 20 patients (14.8%) for hyperalgesic renal colic. We used a 30F nephroscope and ballistic energy in all cases. The method of urine drainage was via a JJ stent in 279 cases (87%). However, 35 patients (11%) were drained via a nephrostomy tube, and 2% (6 patients) required dual drainage. The JJ stent was removed after a period of 30.2 ± 19 days, while the nephrostomy tube was removed on average after 5.7 ± 4 days. A postoperative infectious complication (Clavien–Dindo classification grade ≥ 2) occurred in 59 patients (18.43%). Among these complications, 21 patients (35.6%) had acute pyelonephritis, 31 patients (52.5%) developed sepsis, and 7 patients (11.9%) experienced septic shock. These patients were treated with antibiotics after samples were taken for bacteriological purposes for an average duration of 15 ± 3 days. Eight patients required the use of vasopressors. Among these 59 patients, 23 (39%) underwent preoperative drainage with a double J stent. There was no statistically significant difference between preoperative drainage and the occurrence of infectious complications (*p* = 0.22). Significant blood loss was noted in 57 patients (17.8%). The average drop in hemoglobin was 2.65 ± 1.8 g/dl. Tranexamic acid was required in 20 cases (35.1%) and transfusions in 38 cases (67%), with 3 patients requiring more than four units of packed red blood cells. No patient died from a bleeding complication. The median length of stay was 1 day (1–6). Patients of intermediate or severe frailty were more likely to exhibit postoperative sepsis (*p* = 0.042), significant blood loss (*p* = 0.036) and require intensive care units admissions (*p* = 0.0015). Frail patients had a longer hospital length of stay (*p* < 0.001) and tended to require reoperation (*p* = 0.001), and unplanned readmission (*p* = 0.02) **(**Table 2**)**. Frailty class was found to exhibit a positive correlation with American Society of Anesthesiologists score (*r*^2^ = 0.78, *p* < 0.0001). Patients with higher American Society of Anesthesiologists score were more likely to exhibit postoperative sepsis (*p* = 0.02), significant blood loss (*p* = 0.01), and had a longer hospital length of stay (*p* = 0.04). Postoperative mechanical antithrombotic prophylaxis was used in 72 patients (22.5%) at high risk of thromboembolism.

**Table 1:** Baseline characteristics

Variables	Not Frail	Intermediate	Severely frail	*p*-value
	*N* = 173	*N* = 57	*N* = 90	
Mean age (Years)	47 ± 13	56 ± 11	64 ± 9	0.002
Gender, *N* (%)				0.2
Male	92 (53.1)	32 (56.1)	38 (42.2)	
Female	81 (46.8)	25 (43.9)	52 (57.8)	
Current smoker, *N* (%)	24 (13.9)	8 (14.03)	10 (11.1)	0.67
Component of mFI-5, *N* (%)				
Diabetes mellitus	0	21 (36.8)	35 (38.9)	< 0.001
Congestive heart failure	0	8 (14)	15 (16.7)	< 0.001
Hypertension requiring medication	0	20 (35)	42 (46.7)	< 0.001
Totally or partially dependent functional health status	0	2 (3.5)	3 (3.4)	< 0.001
Chronic obstructive pulmonary disease	0	6 (10.5)	9 (10)	< 0.001
Mean body mass index	22 ± 8	26 ± 5	28 ± 6	0.4
Laterality of procedure, *N* (%)				0.73
Right	89 (51.4)	33 (57.9)	49 (54.4)	
Left	84 (48.6)	24 (42.1)	41 (45.6)	
Prior kidney surgery, *N* (%)	8 (4.6)	4 (7)	4 (4.5)	0.07
Chronic Kidney Disease, *N* (%)	4 (2.3)	15 (26.3)	24 (26.7)	< 0.001
Presence of a staghorn calculus, *N* (%)	24 (13.9)	10 (17.5)	11 (12.3)	0.4
Median number of Stone, *N* (IQR)	1 (1–3)	2 (1–4)	2 (1–3)	0.1
Mean Stone Burden (mm)	28.6 ± 3	35.2 ± 2	32.8 ± 4	0.68
Preoperative hydronephrosis, *N* (%)	125 (72.3)	34 (60)	72 (80)	0.2
Location, *N* (%)				0.5
Upper	33 (19)	10 (17.6)	18 (20)	
Middle	21 (12.2)	4 (7)	6 (6.7)	
Lower	29 (16.8)	7 (12.2)	12 (13.3)	
Renal pelvis	90 (52)	36 (63.2)	54 (60)	
ASA Score, *N* (%)				0.001
ASA 1–2	150 (86.7)	26 (45.6)	33 (36.7)	
ASA 3–4	23 (13.3)	31 (54.4)	57 (63.3)	
Number of dilations	1	1.3	1.5	0.9
Mean surgery time (Min)	90 ± 15	100 ± 20	80 ± 10	0.06
Mean preoperative hemoglobin (g/dl)	14.2 ± 1	11.5 ± 2	9.7 ± 1	0.03

**Table 2:** Association of complication with frailty class

Variables	Not frail	Intermediate	Severely frail	*p*- value
	*N* = 173	*N* = 57	*N* = 90	
Infectious complications (Clavien–Dindo classification grade ≥ 2), *N* (%)				
Urinary tract infection	10 (4.62)	5 (8.77)	6 (6.7)	0.6
Sepsis	4 (2.3)	10 (17.6)	17 (19)	0.042
Septic shock	2 (1.15)	3 (5.26)	2 (2.3)	0.4
Significant blood loss	10 (5.78)	15 (26.3)	32 (35.6)	0.036
30 days post-operative complication				
Median length of stay, days (IQR)	1 (1–2)	3 (1–5)	4 (2–6)	< 0.001
Ancillary procedure, *N* (%)	6 (3.4)	11 (19.3)	19 (21.2)	0.001
Secondary readmission, *N* (%)	7 (4)	17 (30)	23 (26)	0.02
Intensive care units admissions, *N* (%)	1 (0.5)	7 (12.3)	15 (17)	0.0015

## Discussion

This study assesses the mFI-5 as a predictor for postoperative outcomes following PCNL.

### The history of the mFI-5 in urological surgery

Multiple past studies have used frailty indices to illustrate the impact of preoperative frailty scores on postoperative outcomes across all surgical specialties [9, 10]. Frailty is becoming more commonly employed as an indicator of physical readiness for surgery in urological clinical research, particularly within the field of urological oncology [11]. Taylor [12] employed the mFI-5 to assess its impact on postoperative morbidity and health care resource utilization following major urologic oncologic surgeries. They found that patients with an mFI ≥ 3 had twice the risk of increased health care resource utilization compared to those with an mFI of 0, when examining all urologic oncology operations. More recent investigations have employed the Hopkins Frailty Index in retrospective analyses to forecast post-operative results following radical cystectomy [13]. These studies have highlighted connections between frailty and outcomes such as intensive care unit admission, peri-operative mortality, and prolonged hospital stays [13]. The mFI-5 is a well-defined and easily ascertainable predictor of postoperative outcomes in those undergoing minimally invasive partial nephrectomy for localized renal cancer and could be used preoperatively to identify and counsel those at increased risk for morbidity [14].

### Factors associated with complications after PCNL

Shahait [15] retrospectively determined that a 5-mFI score of ≥ 2 correlated with elevated Clavien–Dindo grade complications, heightened risk of extended hospitalization, and early postoperative mortality in patients who underwent radical prostatectomy for prostate cancer. This is the first study to utilize mFI-5 to examine complications of PCNL. Lorenzo [16] showed that stone size, insulin dependent diabetes, and female sex were independently associated with increased risk of Infectious complications following PCNL. A history of diabetes and renal anomalies were associated with developing delayed post-PCNL hemorrhage [17]. According to the study by Açıkgöz [18], advanced age, high body mass index, and the presence of chronic obstructive pulmonary disease were risk factors for pulmonary complications in PCNL.

### Limitations and perspectives

The mFI-5 is an easy-to-apply scale that does not require any additional equipment. Furthermore, staff can be easily trained to measure the five components included in the mFI-5 at the time of the patient’s arrival, making it a cost-effective and simple means of assessing frailty. Additional prospective studies will be necessary to evaluate the mFI-5 in patients with renal stones who are candidates for percutaneous treatment. This could potentially yield results comparable to previous studies and validate the use of the mFI-5 in routine clinical practice. In addition to using this scoring tool in quality-focused retrospective research, the mFI-5 could transform clinical care in real-time. In the future, it would be desirable to integrate a patient’s mFI-5 score and the associated risks for a surgical procedure into their medical record. With this tool, healthcare professionals can identify at-risk patients and use this data to guide multidisciplinary discussions, thereby enabling interventions to reduce risks both in hospital settings and outpatient care.

## Conclusion

Our study highlights the predictive capability of the mFI-5 in patients who underwent PCNL. The mFI-5 proved to be a significant indicator of severe surgical complications in patients with renal stones treated with PCNL. Moreover, future trials will need to examine in greater depth the concept of preoperative rehabilitation and resilience, with the aim of better preparing patients for surgery by reducing their frailty before proceeding with PCNL. Since the frailty phenotype can respond more favorably to such improvements than underlying chronic medical conditions, such an intervention could become a powerful tool for improving outcomes and preventing post-surgical complications following PCNL.

## Data Availability

No datasets were generated or analyzed during the current study.
